# Influence of Alcohol on Muscular Work

**Published:** 1899-11

**Authors:** 


### Influence of Alcohol on Muscular Work.

Destree (*Quarterly Journal of Inebriety*) has made a number of experiments to determine whether more work can be accomplished with alcohol than without it. The results obtained were uniform, and clearly showed that:

1. Alcohol has a favorable effect on the work product whether the muscle is weary or not.

2. This favorable effect appears almost immediately, but is very transitory.

3. Immediately afterward alcohol has a very decided paralyzing effect. About a half hour after taking alcohol the muscular power reaches a maximum that subsequent doses increase with difficulty.

4. The paralyzing effect of alcohol outweighs the momentary stimulation, so that the total work product obtained with the use of alcohol is less than that obtained without it. In other words, alcohol is a deceptive means of dulling the sense of fatigue, but its action is momentary, and in the end injurious, the paralyzing effect upon the nervous system increasing rapidly, and with such force that any momentary good effect cannot counterbalance it. Similar experiments with tea, coffee and kola show that the stimulating effect of these drugs, while less marked than that of alcohol, is continued longer, and is not followed by a paralyzing effect, as is the case with alcohol.—*Charlotte Med. Jour.*

